# Xanthine Oxidase Inhibitors for Improving Renal Function in Chronic Kidney Disease Patients: An Updated Systematic Review and Meta-Analysis

**DOI:** 10.3390/ijms18112283

**Published:** 2017-10-31

**Authors:** Anna Pisano, Valeria Cernaro, Guido Gembillo, Graziella D’Arrigo, Michele Buemi, Davide Bolignano

**Affiliations:** 1 CNR-Institute of Clinical Physiology, 89124 Reggio Calabria, Italy; pisanoanna@hotmail.it (A.P.); g.darrigostat@tin.it (G.D.); 2 Chair of Nephrology, Department of Clinical and Experimental Medicine, University of Messina, 98122 Messina, Italy; valecern82@virgilio.it (V.C.); guidogembillo@live.it (G.G.); buemim@unime.it (M.B.)

**Keywords:** xanthine oxidase inhibitors, allopurinol, febuxostat, topiroxostat, chronic kidney disease, end-stage kidney disease

## Abstract

Background: Accruing evidence suggests that Xanthine Oxidase inhibitors (XOis) may bring direct renal benefits, besides those related to their hypo-uricemic effect. We hence aimed at performing a systematic review of randomized controlled trials (RCTs) to verify if treatment with XOis may improve renal outcomes in individuals with chronic kidney disease (CKD). Methods: Ovid-MEDLINE, PubMed and CENTRAL databases were searched for RCTs comparing any XOi to standard therapy or placebo. The primary endpoint of interest was progression to End-Stage Kidney Disease (ESKD); secondary endpoints were changes in serum creatinine, glomerular filtration rate (eGFR), proteinuria and albuminuria. Results: XOis treatment significantly reduced the risk of ESKD compared to the control (3 studies, 204 pts; RR = 0.42; 95% CI, 0.22, 0.80) and also improved eGFR in data pooled from RCTs with long follow-up times (>3 mo.) (4 studies, 357 pts; mean difference (MD) 6.82 mL/min/1.73 m^2^; 95% CI, 3.50, 10.15) and high methodological quality (blind design) (3 studies, 400 pts; MD 2.61 mL/min/1.73 m^2^; 95% CI, 0.23, 4.99). Conversely, no definite effects were apparently noticed on serum creatinine, proteinuria and albuminuria. Conclusions: XOis may represent a promising tool for retarding disease progression in CKD patients. Future trials are awaited to confirm the generalizability of these findings to the whole CKD population.

## 1. Introduction

The search for alternative strategies to prevent chronic kidney disease (CKD) progression is still an open challenge. In daily practice, currently recommended approaches focusing on lifestyle and dietary modifications, as well as on blood pressure and proteinuria management by renin-angiotensin-aldosterone system (RAAS) antagonists, often fail to produce stable benefits in the long term, particularly in high risk populations [1]. As a result, the rate of individuals with CKD who progress to end-stage kidney disease (ESKD) requiring chronic dialysis remains dramatically high.

A large body of mechanistic and clinical evidence nowadays point at uric acid as a potential therapeutic target for slowing down CKD progression [2]. Gouty patients and even individuals with asymptomatic hyperuricemia have a sustained risk of developing future renal damage; similarly, in patients with overt CKD, steadily elevated uric acid levels may contribute to worsening renal function [3].

In view of their good efficacy and long-term proven safety, xanthine oxidase inhibitors (XOis) currently represent the first-choice treatment of hyperuricemia associated with various diseases, including CKD [4]. In the latter, XOi administration may also ameliorate renal damage, not only by reducing circulating uric acid levels (indirect benefit), but also through various mechanisms at the kidney level (direct benefits), including the reduction of inflammation and oxidative stress and the prevention of glomerular hypertension, afferent arteriolar thickening and ischemic renal histologic changes [5,6,7,8].

Notwithstanding such strong biological premises and a wealth of positive experimental and uncontrolled clinical studies, previously published meta-analyses of randomized controlled trials (RCTs) focusing on Allopurinol showed no effects or only slight improvements in renal function in individuals with overt CKD receiving this therapy [9,10,11].

In the last few years, however, new randomized controlled trials (RCTs) came out, providing novel evidence of renal benefits of XOis. Of note, the majority of these new studies employed Febuxostat and Topiroxostat, two “second-generation” XOis that are considered to be endowed with a more powerful reno-protective potential than Allopurinol [12].

This recently accrued new body of evidence calls for the necessity of a new, comprehensive systematic review and meta-analysis in order to clarify whether XOis could indeed be useful for improving renal outcomes in the CKD population.

## 2. Results

### 2.1. Search Results

**Figure 1:**
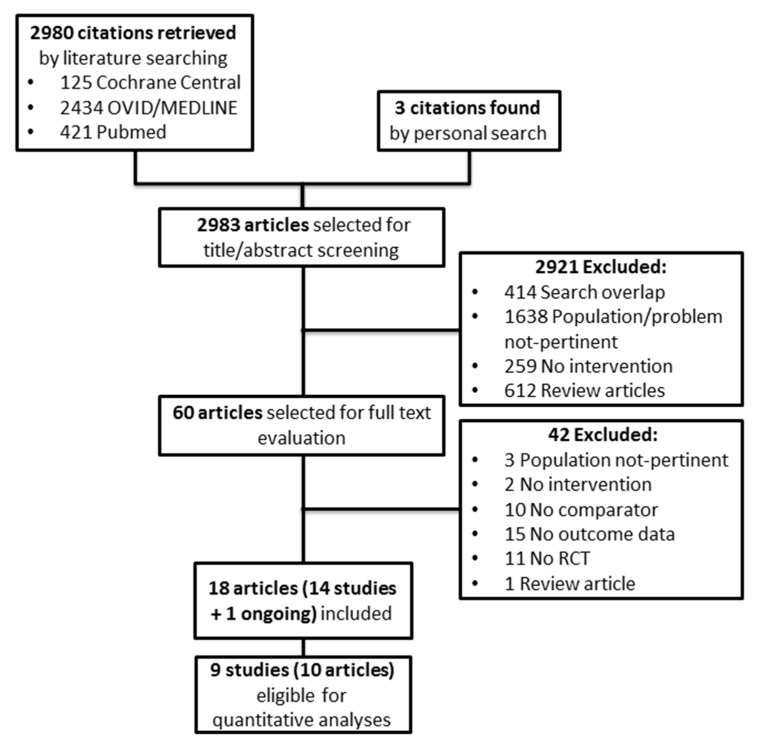
Study selection flow. RCT: randomized controlled trial.

Figure 1 shows the flow diagram of the study selection process. Two thousand nine hundred and eighty potentially relevant references were initially found. Three additional citations were added by a personal search. By screening titles and abstracts, a total of 2921 citations were excluded for various reasons (search overlap, study population or intervention not pertinent, review articles or other topics). Amongst the 60 studies selected for full text examination, 42 studies were excluded because: (1) non-randomized controlled trials (*n* = 11); (2) review articles (*n* = 1); (3) dealing with the wrong population (*n* = 3) or intervention/comparator (*n* = 12); (4) not providing data on the outcomes of interest (*n* = 15).

A total of 18 articles referring to 14 studies (1096 participants) and one ongoing trial were finally included in the review.

Nine randomized trials (695 participants) provided suitable numerical data on the outcomes of interest and were included in cumulative meta-analyses. The main characteristics of the studies reviewed are described in Table 1.

### 2.2. Study Characteristics

All the studies reviewed [13,14,15,16,17,18,19,20,22,23,24,25,26,27] had a parallel design. Three studies were multicenter [17,18,27]. The number of participants ranged from 40 [14,16] to 179 [18]. All trials reviewed [13,14,15,16,17,18,19,20,22,23,24,25,26,27] enrolled hyperuricemic (uric acid ≥ 6 mg/dL) CKD patients. Baseline uric acid levels ranged from ~6.2 [14] to 10.5 mg/dL [27]. Study participants had early renal failure (NKF KDOQI stage 2) in two RCTs [16,18] and mild-to-moderate (stage 3–4) CKD in nine [13,15,17,19,20,22,23,24,25]. One study [27] enrolled individuals with moderate-to-severe (stage 4–5) CKD. The prevalence of diabetes was available in eight studies [13,14,17,22,24,25,26,27], ranging from 21.5% [17] to 100% [14,26]. The mean age of patients ranged from ~40 [16] to 72.2 years [15]. Male gender ranged from 45% [14] to 100% [18]. Study follow-up varied from 4 weeks [18] to 84 months [20].

The type of XOi employed was Allopurinol in nine studies [13,14,15,16,18,19,20,22,23], Febuxostat in six [18,23,24,25,26,27] and Topiroxostat in one [17]. Drug intervention was compared to a placebo [14,15,17,18,24,26,27] or standard therapy [13,16,19,20,22,23,25]. Two RCTs [18,23] tested the effect of both Allopurinol and Febuxostat vs. the control. The daily dose of Allopurinol administered ranged from 100 [14,20] to 300 mg/day [15,16,18,22,23]. The dose of Febuxostat varied from 30 mg/twice a day [27] to 120 mg/day [18]. Saag et al. [27] tested Febuxostat at two different dose regimens (30 mg/twice a day, 40–80 mg/day). In the study conducted by Hosoya et al. [17], patients received Topiroxostat at a daily dose of 160 mg/day. End-of treatment uric acid levels ranged from 3.9 [26] to 6.6 mg/dL [20].

### 2.3. Risk of Bias

Risk of bias of randomized controlled trials is summarized in Table 2. Information on the random sequence generation and allocation concealment was reported in seven [13,16,20,22,24,25,26] and four studies [16,24,25,27], respectively. Seven RCTs [14,15,17,18,24,26,27] were double blind, six studies were open label [13,16,19,22,23,25] and only one [20] had a single-blind design. Only four [20,24,25,26] specifically provided information on blinding of the outcome assessors. Attrition bias was low in nine studies [13,16,17,18,20,24,25,26,27] and unclear in four [14,19,22,23]; in the RCT reported by Kao et al. [15], the overall drop-out rate was 25%. Reporting bias was low in all studies [13,14,15,16,17,18,19,20,22,23,24,25,26,27]. Risk of funding bias was potentially high in two studies [17,27] while two other studies specifically declared any sponsor involvement [24,26]. No other potential source of bias was apparently present in the remaining studies [13,14,15,16,18,19,20,22,23,25].

### 2.4. Outcome Data

Data on the combined endpoint of progression to ESKD (serum creatinine doubling, eGFR decrease ≥50% or need for dialysis therapy) was available in only three RCTs [13,16,20]. Five studies provided data on serum creatinine change from baseline values [13,14,18,25,27]; information on change in creatinine clearance/eGFR was reported by 12 studies [15,16,17,18,19,20,22,23,24,25,26,27]. End of treatment proteinuria and albuminuria was analyzed in six [13,14,15,16,22,25] and four RCTs [17,23,25,26], respectively.

### 2.5. Effects of Xanthine Oxidase Inhibitors on Progression to ESKD

**Figure 2:**
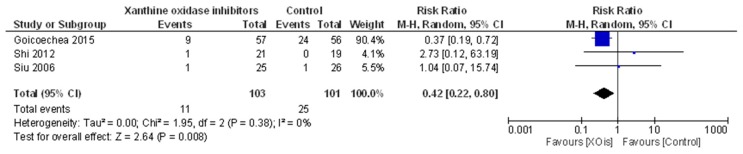
Effects of XOis vs. control on progression to end-stage kidney disease (ESKD).

In a pooled meta-analysis of three RCTs (204 individuals) [13,16,20], XOis reduced the risk of the combined endpoint of progression to ESKD with respect to the control (RR = 0.42; 95% CI, 0.22, 0.80; Figure 2), with no heterogeneity in the analysis (χ^2^ = 1.95, *p* = 0.38; I^2^ = 0%). The quality of the body of evidence for this outcome (GRADE) was high (Table 3).

### 2.6. Effects of Xanthine Oxidase Inhibitors on Secondary Outcomes

#### 2.6.1. Serum Creatinine

**Figure 3:**
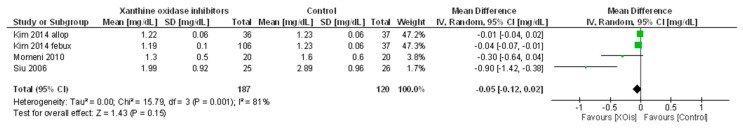
Effects of XOis vs. control on serum creatinine.

Two studies [25,27] reported no concrete effects of Febuxostat on serum creatinine with respect to the control. These observations were in line with a pooled meta-analysis of three RCTs (4 intervention arms; 270 individuals) [13,14,18], showing no significant change in serum creatinine after treatment with XOis versus the control (MD −0.05 mg/dL; 95% CI, −0.12, 0.02; Figure 3). This analysis was affected by high heterogeneity (χ^2^ = 15.79, *p* = 0.001; I^2^ = 81%) that was significantly reduced (I^2^ = 58%) after excluding the only study with an open label design [13]. The quality of the body of evidence for this outcome (GRADE) was very low after being downgraded for high inconsistency and indirectness (applicability in study population/intervention/follow-up/study design) (Table 3).

Visual inspection of the funnel plot and the Egger’s regression test (*p* = 0.13) indicate that the presence of publication bias was unlikely (Supplementary Figure S1a).

#### 2.6.2. Renal Function

In one trial [23], eGFR significantly increased after Febuxostat administration, as compared to standard therapy. Conversely, four studies [15,17,26,27] did not report significant differences in eGFR after treatment with XOis or placebo.

**Figure 4:**
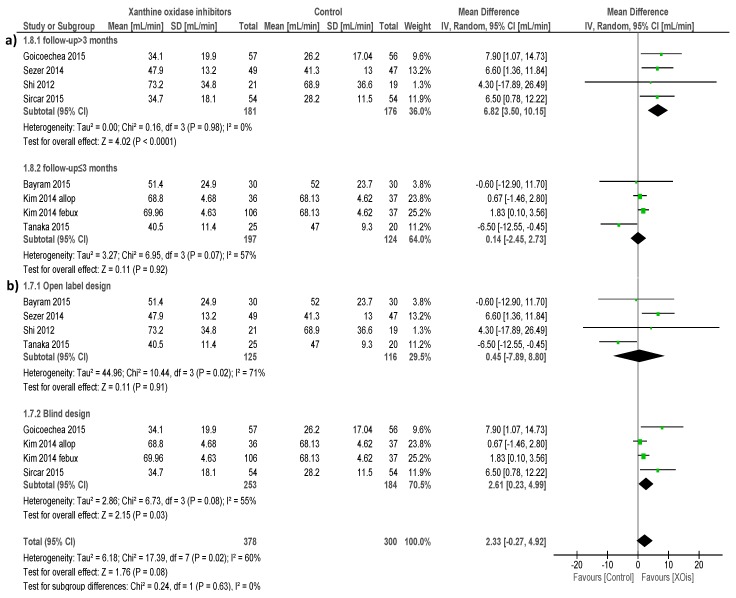
Effects of XOis vs. the control on renal function.

This latter observation was in agreement with findings from a cumulative meta-analysis of seven RCTs (8 intervention arms; 641 individuals) [16,18,19,20,22,24,25], showing no apparent effect of XOi administration on renal function compared with the control (MD 2.33 mL/min/1.73 m^2^; 95% CI, −0.27, 4.92; Figure 4). Visual inspection of the funnel plot and the Egger’s regression test (*p* = 0.63) show absence of publication bias (Supplementary Figure S1b). The GRADE quality of this analysis was very low after downgrading for high inconsistency and indirectness (applicability in study population/intervention/follow-up/study design) (Table 3), and a mild level of heterogeneity was present (χ^2^ = 17.39, *p* = 0.02; I^2^ = 60%). Study stratification by CKD stage of participants, baseline and end-of-treatment uric acid levels, type of XOi administered or study design (blind vs open label) had no impact on such heterogeneity.

Conversely, variable follow-up length across studies appeared to be the major determinant of heterogeneity, as this was fully nullified by sensitivity analyses including only studies with longer duration (>3 months) (χ^2^ = 0.16, *p* = 0.98; I^2^ = 0%). It was very interesting, that when focusing on such long-term studies, the impact of XOi treatment on eGFR also became significantly positive (4 studies, 357 individuals; MD 6.82 mL/min/1.73 m^2^; 95% CI, 3.50, 10.15; Figure 4a) compared with the control.

Given the absence of inconsistency and the limited indirectness, the GRADE quality of this sub analysis increased to moderate. In subgroup analyses restricted to studies with a blind design, benefits of XOis over the control with respect to renal function remained significant (3 studies, 400 individuals; MD 2.61 mL/min/1.73 m^2^; 95% CI, 0.23, 4.99; Figure 4b), although the quality of this analysis was downgraded to low (presence of inconsistency and indirect applicability in study intervention).

#### 2.6.3. Proteinuria

Tanaka et al. [25] reported a significant reduction in the urinary protein/creatinine ratio in individuals on Febuxostat therapy vs. standard therapy (−0.36 ± 0.66 vs. 0.07 ± 0.38 g/g; *p* = 0.018).

Conversely, in another trial [15], Allopurinol had no effects over the placebo on proteinuria excretion.

**Figure 5:**
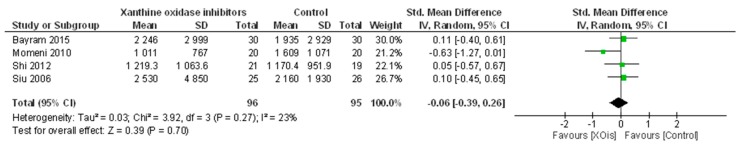
Effects of XOis vs. the control treatment on proteinuria.

This latter observation was consistent with data from a meta-analysis of four RCTs (191 individuals) [13,14,16,22], showing no significant change in proteinuria levels in the active arm compared with the control (SMD −0.06; 95% CI, −0.39, 0.26; Figure 5). This analysis had a low level of heterogeneity (χ^2^ = 3.92, *p* = 0.27; I^2^ = 23%). Publication bias was very unlikely according to visual inspection of the funnel plot and results from Egger’s regression test (*p* = 0.30) (Supplementary Figure S1c). The quality of the body of evidence for this outcome (GRADE) was high (Table 3).

#### 2.6.4. Albuminuria

Three single studies [17,23,25] reported a significant reduction of urinary albumin/creatinine levels in individuals taking XOis compared to the control. In these studies, data were reported in a format that was not suitable to be pooled in a cumulative meta-analysis. On the contrary, in another trial [26], no differences were found in this parameter after Febuxostat or placebo treatment.

## 3. Discussion

This systematic review has been performed with the purpose of clarifying whether XOi treatment may exert benefits on renal outcomes in CKD patients, besides their acknowledged utility and efficacy in reducing circulating uric acid levels.

Indeed, a wealth of mechanistic and experimental evidence previously indicated that this drug class may be endowed with some nephroprotective effects. This ranges from the improvement of oxidative stress by reducing reactive oxygen species generation at the kidney level [28] to the amelioration of endothelial dysfunction and inflammation [29].

As intra-renal oxidative stress exacerbates smooth muscle cell proliferation of the afferent arterioles and promotes renin-angiotensin system activation, XOis would also improve kidney micro-perfusion, thereby preventing glomerular hypertension and ischemic renal histologic changes [8,30]. Such a biological background would give the rationale for explaining a series of clinical benefits, including the improvement in proteinuria, hypertension and renal function, which have been reported by various observational and interventional studies [31].

Three other meta-analyses already approached this issue in the past, providing scant or indefinite conclusions and partial disagreement among findings reported [9,10,11].

We therefore felt it necessary to perform a new, updated systematic analysis of the available evidence, also in light of a series of new RCTs that have been finalized in the last few years on the same topic. Some of these trials provided novel evidence of the benefits of XOis and tested the effects of new-generation XOis (Febuxostat, Topiroxostat) that were not considered by some previous systematic reviews because they were not yet available at that time.

From a general point of view, the findings obtained in our review seem to support the hypothesis that XOis can improve disease course in individuals with non-advanced CKD.

In particular, in a pooled analysis of three studies including 204 participants, treatment with such drugs was associated with a significant reduction (RR = 0.42; 95% CI, 0.22, 0.80) in the risk of progression to a combined ESKD endpoint (encompassing the most widely used binary criteria to define ESKD occurrence), as compared with the control. Of note, although relying on a few studies, this analysis had null heterogeneity and the corresponding body of evidence (GRADE) was of high quality, according to a validated 5-item list of methodological assessment (absence of study limitations, inconsistency of effect, imprecision, indirectness and publication bias) [32]. This latter observation may indicate that further research is unlikely to change the confidence in the estimate of effect.

Renal benefits of XOis in CKD patients were somewhat confirmed when looking at “continuous” parameters of kidney function, although under particular conditions.

In fact, an overall cumulative analysis of seven trials enrolling a total of 641 individuals did not show evidence of any significant impact of XOis, compared to the control, on estimated glomerular filtration rate (eGFR). This apparent lack of effect was in line with data from four single RCTs (not suitable to be included in the same meta-analysis) [15,17,26,27] as well as with findings published in two previous systematic reviews [9,11].

The results from this analysis, however, could be considered poorly reliable “such as they are”, given the presence of relevant heterogeneity (60%) and the very low GRADE quality of the body of evidence for high inconsistency and indirectness. When looking at potential sources of heterogeneity by exploratory subgroup analyses, we found that duration of treatment (study length) was the main factor responsible for this condition. Of note, such separate analysis also revealed the capacity of XOis to produce a clinically significant improvement in eGFR values (MD 6.82 mL/min/1.73 m^2^; 95% CI, 3.50, 10.15) if the observation is restricted to long-term studies only. This finding is not particularly surprising, bearing in mind that stable improvements in renal function by therapies directly targeting kidney function are usually related to hemodynamic adaptations and parenchymal/histological modifications that need more than few weeks to manifest. Accordingly, in a previous meta-analysis, similar although less remarkable benefits on eGFR (MD 3.2 mL/min/1.73 m^2^) were confined only to inception analyses considering trials longer than 3 months [10]. Of note, we also noticed a slightly positive effect of XOis on renal function in subgroup analyses restricted to blind trials (MD 2.61 mL/min/1.73 m^2^; 95% CI, 0.23, 4.99); this would support the need to minimize potential detection and performance bias of future trials by making use of a blind design in order to avoid a confounding effect on treatment efficacy.

In a pooled analysis of three studies (270 individuals), XOis had no definite effects on serum creatinine, an observation in line with findings from two other single trials [25,27] and with a previously published systematic review [11]. Although this observation might contradict the above-reported positive effects on eGFR, the true significance remains questionable given the partially unexplained heterogeneity and the very low quality of the body of evidence for high inconsistency and indirectness.

In a high quality, low-heterogeneity analysis pooling of data from four RCTs, no tangible benefits of XOis over the control were evidenced on proteinuria levels. This result confirms findings reported from previous meta-analyses [9,10,11] and from another single trial of Allopurinol [15]. Conversely, XOi treatment seemed to be effective in improving urinary albumin excretion in single data obtained from three RCTs [17,23,25]. Unfortunately, as the information from these trials was provided in a format not suitable to be pooled in cumulative analyses, the question as to whether these drugs may also improve early renal damage remains cannot be answered in a definite manner.

Our paper has a series of strengths and limitations that deserve mentioning. This review follows all current best methodological standards for systematic reviews including a pre-published protocol, a thorough literature search of multiple databases by focused, high sensitive search strategies and a systematic approach to study selection, data extraction, cumulative analyses and bias and outcome quality assessment. The key limitations of this review are represented by the few number of trials suitable to be included in cumulative analyses and the strength and quality of information available from single studies. Despite good homogeneity across studies in terms of population characteristics (e.g., CKD stage, baseline and end-of-treatment uric acid levels, co-morbidities, etc.), a substantial percentage of the included trials had a questionable (open label) design, were single-center, enrolled few participants and were of short to very-short duration. Only a few RCTs looked specifically at solid outcomes, such as the need for dialysis or kidney transplantation, while the remaining were mostly powered to catch differences in surrogate endpoints. No less important, information on the effects of XOis on early renal damage (albuminuria) was sparse or lacking.

The low number of studies finally included in the meta-analyses prevented the possibility of performing more complex investigations, such as additional subgroup or meta-regression analyses, as initially pre-planned, in order to identify all potential treatment-effect modifiers. Although we were able to explain major sources of heterogeneity for relevant outcome analyses and to identify duration of treatment and study quality as major determinants of response to treatment, the question as to whether the beneficial effects of XOis on renal function can be generalized to the whole CKD population remains to be answered.

In conclusion, there is cumulative evidence suggesting that, besides the hypo-uricemic effect, long-term treatment with Xanthine Oxidase inhibitors may bring reno-protective benefits in individuals with non-advanced chronic kidney disease. Future trials targeting solid rather than surrogate renal endpoints (e.g., ESKD) that are designed according to the highest methodological standards (double or triple-blind) are needed to support this observation further. The question as to whether administration of these agents may also positively impact early clinical damage remains to be solved by upcoming research.

## 4. Materials and Methods

This review follows Preferred Reporting Items for Systematic Reviews and Meta-Analyses (PRISMA) guidelines [33] for reporting in systematic reviews and meta-analysis and was conducted according to a previously published protocol [34].

### 4.1. Data Source and Search Strategy

Ovid-MEDLINE, PubMed and CENTRAL databases were searched for articles without time or language restriction up to 28 June 2017 using focused, highly sensitive search strategies (Supplementary Table S1). References from relevant studies and reviews were screened for additional articles. The search was designed and performed by two Authors (Davide Bolignano, Anna Pisano).

### 4.2. Study Selection and Data Extraction

We aimed at including any RCT or quasi-RCT (trials in which allocation to treatment was made by alternation, use of alternate medical records, date of birth or other expected methods) providing evidence on potential benefits on kidney function/damage of first and second generation XOis in patients with CKD.

Studies were considered regardless of dosage or duration of administration of XOis and without follow-up duration restrictions. The type of comparator was a placebo or standard treatment.

The presence of CKD was defined according to the National Kidney Foundation-Kidney Disease Outcomes Quality Initiative (NKF KDOQI) guidelines [35] by a reduced glomerular filtration rate (GFR) <90 mL/min/1.73 m^2^ or by the persistence of urinary abnormalities such as albuminuria, proteinuria or hematuria in subjects with GFR ≥90 mL/min/1.73 m^2^.

The primary endpoint of interest was progression to End-Stage Kidney disease (ESKD) defined as one of the following events: serum creatinine doubling, eGFR decrease ≥50%, need for dialysis therapy or kidney transplantation. Secondary outcomes were changes in serum creatinine, renal function (creatinine clearance/eGFR), proteinuria and albuminuria.

Studies were excluded for the following reasons: (1) they dealt with hyperuricemic/gouty individuals without manifested CKD or on chronic renal replacement therapy (e.g., hemodialysis or peritoneal dialysis); (2) the did not provide short- or long-term data on the outcomes of interest; (3) they reported on treatment with other drugs endowed with hypouricemic effects not belonging to the XOi class (e.g., Benzbromarone, Rasburicase, Losartan); (4) they were non-randomized controlled trials.

Titles and abstracts were screened independently by two authors (Valeria Cernaro, Guido Gembillo) who discarded studies that were not pertinent to the topic. Non-randomized studies, reviews, editorials, letters and studies performed on children (age < 18) were excluded from qualitative analyses but screened for potential additional references. Two Authors (Anna Pisano, Valeria Cernaro) independently assessed the retrieved abstracts and the full text of these studies to determine eligibility according to the inclusion/exclusion criteria.

A third reviewer (DB) solved possible discrepancies on study judgments. Data extraction and analysis were performed by two reviewers (Anna Pisano, Valeria Cernaro) and independently verified by another (Guido Gembillo).

### 4.3. Data Analysis

Cumulative meta-analyses were performed for outcomes in which data were provided in a suitable and consistent format and by more than two studies. In order to maximize the information provided to readers, data on outcomes reported by single studies or in a descriptive way were reported narratively. The effects of treatment on continuous variables were assessed as the mean difference (MD) or standardized mean difference (SMD), as appropriate. The relative risk (RR) was calculated for dichotomous outcomes. Data were pooled using the random-effects model. To ensure robustness of the model and susceptibility to outliers, pooled data were also analyzed with the fixed-effects model. Heterogeneity was assessed by the χ-squared test on *N* − 1 degrees of freedom, with an alpha of 0.05 considered for statistical significance and the Cochrane-I-squared statistic [36]. I^2^ values of 25%, 50% and 75% were considered to correspond to low, medium and high levels of heterogeneity, respectively. Sources of heterogeneity, for identifying possible effect modifiers on the pooled analyses, were explored by sensitivity analyses according to: population characteristics (e.g., severity of CKD), duration of follow-up, study design and quality, type of intervention employed and baseline/end of treatment uric acid levels.

Given the overall paucity of studies looking at similar outcomes, we could not perform meta-regression analyses, as originally planned in the review protocol.

Publication bias was investigated by Egger’s regression test and by visual inspection of funnel plots. Statistical analyses were performed by two Authors (AP, GD) using Review Manager (RevMan; Version 5.3. Copenhagen: The Nordic Cochrane Centre, The Cochrane Collaboration, 2014) and Stata/IC (Version 13.1, StataCorp LP, College Station, TX, USA).

### 4.4. Risk of Bias (Quality) Assessment

Likelihood of bias in the single RCTs was evaluated by using the checklist developed by the Cochrane Renal Group, which considers the presence of potential selection bias (random sequence generation and allocation concealment), performance bias (blinding of investigators and participants), detection bias (blinding of outcome assessors), attrition bias (incomplete outcome data), reporting bias (selective reporting) and possible other sources of bias (e.g., funding bias).

### 4.5. Summary of Findings and Quality of the Evidence

A “Summary of findings” table summarizing pooled evidence for the main outcomes was constructed according to the GRADE method [32]. The five GRADE considerations (study limitations, consistency of effect, imprecision, indirectness and publication bias) were taken into account to assess the quality of a body of evidence for the main pre-specified outcomes. All decisions to downgrade or upgrade the quality of studies were justified using footnotes, and comments were made, when appropriate, to help readers’ understanding of the review.

## Figures and Tables

**Table 1:** Summary of main characteristics and findings of the RCTs reviewed.

Study, Year (Ref)	Study Population	Population Characteristics	Duration	Intervention	Comparator	Outcome(s)	Results	Notes
Siu et al., 2006 [13]	-Hyperuricemic, mild to moderate CKD patients -Exclusion criteria: history of gouty arthritis, renal stones and advanced CKD, use of Allopurinol or Azathioprine, Allopurinol hypersensitivity, pregnancy or lactation	-*N* = 51 -Age (yr) = ~48.2 -Weight (kg) = ~68 -DM (%) = ~25.5 -Hypertension (%) = ~78.5 -SBP (mmHg) = ~136.5 -DBP (mmHg) = ~75 -Uric acid (mg/dL) = ~9.83 -SCr (mg/dL) = ~1.75 -Proteinuria (g/d) = ~2.39	12 months	Allopurinol, 100–200 mg/day (*N* = 25)	Standard therapy (*N* = 26)	SCr (mg/dL)	No difference between groups	-Open label -Allopurinol dose was adjusted according to baseline renal function -Antihypertensive, lipid-lowering and steroid drugs were continued during the study -One patient in the Allopurinol group withdrew due to urticarial skin rash; two lost to follow-up in control group -Per-protocol analysis performed
						Proteinuria (g/day)	No difference between groups	
						Need for dialysis	One patient in Allopurinol (1/25) and control group (1/26), respectively	
Momeni et al., 2010 [14]	-Type 2 diabetic patients with nephropathy (proteinuria ≥ 500 mg/d, SCr < 3 mg/dL) -Exclusion criteria: history of Allopurinol hypersensitivity or past use of Allopurinol for other reasons, SCr > 3 mg/dL or GFR < 25 mL/min, systemic diseases or other causes of proteinuria	-*N* = 40 -Men (%) = 45 -Age (yr) = 57.7 ± 10.5 -Weight (kg) = ~75.4 -BMI (kg/m^2^) = ~27.8 -DM duration (yr) = 12.6 ± 6.7 -SBP (mmHg) = ~146.5 -DBP (mmHg) = ~87.2 -Uric acid (mg/dL) = ~6.2 -SCr (mg/dL) = ~1.4 -Proteinuria (mg/d) = ~1714.5 -Urine Cr (mg/d) = ~1064.5	4 months	Allopurinol, 100 mg/day (*N* = 20)	Placebo (*N* = 20)	SCr (mg/dL)	No difference between groups	-Double blind -Patients continued their concomitant treatment
						Proteinuria (mg/day)	-End of treatment, 1011 ± 767 vs. 1609 ± 1071 in allopurinol vs. placebo group (*p* = 0.049)	
Kao et al., 2011 [15]	-Stage 3 CKD patients with LVH -Exclusion criteria: active gout, LVF with EF < 45%, severe hepatic disease, use of Warfarin, Theophyllin, Allopurinol, Chlorpropamide, immunosuppressive therapy, metastatic malignancy, pregnancy	-*N* = 53 -Men (%) = ~58 -Age (yr) = ~72.2 -SBP (mmHg) = ~142 -DBP (mmHg) = ~72.5 -Uric acid (mmol/L) = ~0.43 -eGFR (mL/min/1.73 m^2^) = ~45 -UPCR (mg/mmol) = ~37.5	9 months	Allopurinol, 300 mg/day (*N* = 27)	Placebo (*N* = 26)	eGFR (mL/min/1.73 m^2^)	No difference between groups	-Double blind -Patients continued their concomitant treatment -3/14 patients withdrew due to rash and arthralgia in Allopurinol group -Per-protocol analysis performed
						UPCR (mg/mmol)	No difference between groups	
Shi et al., 2012 [16]	-Hyperuricemic IgAN patients -Exclusion criteria: active gout, prednisone or immunosuppressive use within the preceding 2 months, ACEIs and/or ARBs use, Allopurinol intolerance, pregnancy	-*N* = 40 -Men (%) = ~55 -Age (yr) = ~40 -SBP (mmHg) = ~140 -DBP (mmHg) = ~87.7 -Hypertension (%) = ~45 -Uric acid (mg/dL) = ~7.9 -SCr (mg/dL) = ~1.35 -eGFR (mL/min) = ~66.5 -UPCR (mg/g) = ~898	6 months	Allopurinol, 100–300 mg/day (*N* = 21)	Standard therapy (*N* = 19)	eGFR (mL/min/1.73 m^2^)	No difference between groups	-Open label -Three patients in the Allopurinol and two patients in control group discontinued the study; no patients were lost to follow-up -ITT analysis performed
						UPCR (mg/g)	No difference between groups	
Hosoya et al., 2014 * [17] Hara et al., 2015 (post-hoc) Jomori et al., 2015 (post-hoc)	-Hyperuricemic stage 3 CKD patients with or without gout -Exclusion criteria: gouty arthritis within 2 weeks before the study, nephrotic syndrome, nephrolithiasis or urolithiasis, hyperuricemia secondary to cancer or other diseases, HbA1c ≥ 8%, severe hypertension, hepatic dysfunction, cancer, pregnancy, breastfeeding, serious heart disease	-*N* = 122 -Men (%) = ~54.5 -Age (yr) = ~63.5 -BMI (kg/m^2^) = ~25.6 -Uric acid (µmol/L) = ~503.8 -DM (%) = ~21.5 -Diabetic nephropathy (%) = ~16.5 -SBP (mmHg) = ~135 -DBP (mmHg) = ~84.5 -eGFR (mL/min/1.73 m^2^) = ~49.2 -UACR (mg/g) = ~35.8	22 weeks	Topiroxostat, 160 mg/day (*N* = 62)	Placebo (*N* = 60)	eGFR (mL/min/1.73 m^2^)	-No difference between groups -No difference between groups when stratifying for DM nephropathy and nephrosclerosis	-Double blind -Topiroxostat and placebo were administered orally for 2 weeks at an initial dose of 40 mg/day, followed by an increase to 80 mg/day for 4 weeks, to 120 mg/day for 8 weeks, and to 160 mg/day for other 8 weeks -Six and five patients, in the Topiroxostat and placebo group, respectively, withdrew from the study due to AEs -ITT analysis performed
						UACR (%)	-Mean percent change -33 (95% CI, −45.0, −20.0) vs. −6 (95% CI, −22.0, 14.0) in Topiroxostat vs. placebo group (*p* = 0.009) -Mean percent change -33.8 vs. +9 (*p* = 0.059) and −44.8 vs. +3.4 (*p* = 0.022), in Topiroxostat vs. placebo group when stratifying for DM nephropathy and nephrosclerosis, respectively	
Kim et al., 2014 [18]	-Gouty patients with early renal function impairment -Exclusion criteria: SCr > 1.5 mg/dL, use of thiazide diuretics or medications containing Aspirin or other salicylates, active liver disease and alcohol intake > 14 drinks/week	-*N* =179 -Men (%) =100 -Age (yr) =~50 -BMI (kg/m^2^) =~25.9 -SBP (mmHg) =~129.7 -DBP (mmHg) =~82.1 -SCr (mg/dL) =~1.2 -eGFR(mL/min/1.73 m^2^) = ~68.6	1 month	Febuxostat, 40 mg/day (*N* = 35) Febuxostat, 80 mg/day (*N* = 35) Febuxostat, 120 mg/day (*N* = 36) Allopurinol, 300 mg/day (*N* = 36)	Placebo (*N* = 37)	SCr (mg/dL)	-End of treatment, 1.19 ± 0.10 vs. 1.23 ± 0.06 in the combined Febuxostat group (*N* = 106) vs. placebo (*p* = 0.007) -No difference between Allopurinol and placebo	-Double blind -Seven patients (placebo = 1, Febuxostat 80 mg/d = 1, Febuxostat 120 mg/d = 2, Allopurinol = 2) missed a follow-up or withdrew prematurely after week 2 -Missing data were analysed by applying the last-observation-carried-forward method
						eGFR (mL/min/1.73 m^2^)	-End of treatment, 69.96 ± 4.63 vs. 68.13 ± 4.62 in the combined Febuxostat group (*N* = 106) vs. placebo (*p* = 0.03) -No difference between Allopurinol and placebo group	
Sezer et al., 2014 [19]	-Stage 3–4 CKD patients -Exclusion criteria: history of Allopurinol intolerance, ongoing Allopurinol treatment, active infections or inflammatory diseases, chronic liver disease and ongoing immunosuppressive therapy	-*N* = 96 -Men (%) = 57 -Age (yr) = 65.3 ± 12.4 -eGFR(mL/min/1.73 m^2^) = ~45.8	12 months	Allopurinol, 1.5 ± 0.8 mg/kg/d (*N* = 49)	Standard therapy (*N* = 47)	eGFR (mL/min/1.73 m^2^)	-End of treatment, mean change 3.3 ± 1.2 vs. −1.3 ± 0.6 in Allopurinol vs. control group (*p* = 0.04)	-Open label -No hematologic alterations or serious adverse events in relation to Allopurinol treatment
Goicoechea et al., 2015 * [20] Goicoechea et al., 2010 [21]	-Moderate CKD patients (eGFR < 60 mL/min/1.73 m^2^) -Exclusion criteria: history of hypersensitivity or past use of Allopurinol, active infections or inflammatory diseases, HIV infection, chronic hepatopathy and use of immunosuppressive therapy	-*N* = 107 -Age (yr) = ~71.7 -SBP (mmHg) = 147 ± 20 -DBP (mmHg) = 77 ± 11 -Uric acid (mg/dL) = ~7.6 -SCr (mg/dL) = ~1.8 -eGFR(mL/min/1.73 m^2^) = ~40 -Urinary albumin (mg/day) = ~36 (median)	84 months	Allopurinol, 100 mg/day (*N* = 56)	Standard therapy (*N* = 51)	eGFR (mL/min/1.73 m^2^)	-End of treatment, 34.1 ± 12.9 vs. 26.2 ± 17.4 in Allopurinol vs. control group	-Single blind -Antihypertensive, lipid-lowering and antiplatelet drugs were continued during the study period -Two patients in Allopurinol group withdrew because of gastrointestinal symptoms -Nine patients in the control and 4 in the Allopurinol group were lost to follow-up -ITT analyses performed
						Need for dialysis	7/57 pts in Allopurinol and 13/56 in control group, respectively	
						eGFR decrease ≥ 50% or SCr doubling	2/57 pts in Allopurinol and 11/56 in control group, respectively	
Bayram et al., 2015 [22]	-Hyperuricemic (uric acid > 5.5 mg/dL) stage 2–4 CKD patients -Exclusion criteria: dialysis, hyperuricemia due to malignancy, peripheral arterial disease, gouty arthritis or history of Allopurinol intolerance, ongoing Allopurinol treatment, active infections or inflammatory diseases	-*N* = 60 -Men (%) = ~46.7 -Age (yr) = ~57.4 -BMI (kg/m^2^) = ~26.8 -SBP (mmHg) = ~133.6 -DBP (mmHg) = ~77.5 -DM (%) = ~57 -Hypertension (%) = 63.3 -Uric acid (mg/dL) = ~7.8 -SCr (mg/dL) = ~2.1 -CrCl (mL/min) = ~49.6 -Proteinuria (mg/d) = ~2136	3 months	Allopurinol, 300 mg/day (*N* = 30)	Standard therapy (*N* = 30)	eGFR (mL/min)	-Significant increase (43.4 ± 20.1 to 51.4 ± 24.9) in the Allopurinol group (*p* = 0.011) -No change in the control group	-Open label -Antihypertensive drugs, lipid-lowering agents and antiplatelet drugs were continued during the study -No adverse effects related to Allopurinol treatment
						Proteinuria (mg/day)	No significant change in the Allopurinol or control group	
Ivanov and Ivanova, 2015 [23]	-Non-diabetic stage 2–3 CKD patients with mild hypertension and no history of gout	-*N* = 56 -eGFR (mL/min) = 54 ± 3	14 months	Allopurinol, 300 mg/day (*N* = 20) Febuxostat, 80 mg/day (*N* = 16)	Standard therapy (*N* = 20)	eGFR (mL/min)	-End of treatment, increase in Febuxostat (+14 ± 3) vs. control group (*p* < 0.01)	-Open label
						Urinary albumin (mg/day)	-End of treatment, decrease in Febuxostat (−138 ± 22) vs. control group (*p* < 0.01)	
Sircar et al., 2015 [24]	-Stage 3–4 CKD patients with asymptomatic hyperuricemia (uric acid ≥ 7 mg/dL) -Exclusion criteria: medication (excluding diuretics) or conditions that may increase uric acid levels such as disorders of primary uric acid metabolism. Autosomal dominant polycystic kidney disease, pregnancy, lactation and symptomatic hyperuricemia	-*N* = 108 -Men (%) = ~70.5 -Age (yr) = ~57.3 -stage 3 CKD (%) = ~47 -stage 4 CKD (%) = ~54 -SBP (mmHg) = ~144 -DBP (mmHg) = ~82.9 -DM (%) = ~37.5 -Hypertension (%) = ~98 -Uric acid (mg/dL) = ~8.6 -SCr (mg/dL) = ~2.2 -eGFR(mL/min/1.73 m^2^) = ~32	6 months	Febuxostat, 40 mg/day (*N* = 54)	Placebo (*N* = 54)	eGFR (mL/min/1.73 m^2^)	End of treatment, 34.7 ± 18.1 vs. 28.2 ± 11.5 in Febuxostat vs. placebo group (*p* = 0.05)	-Double blind -Both groups received antihypertensive agents, including ACEIs or ARBs or diuretics -About 10% of the randomly assigned population withdrew -A modified ITT analysis was performed for efficacy and safety data (*N* = 98) -Two patients in Febuxostat had mild diarrhoea
						eGFR decrease ≥10%	38% vs. 40% in Febuxostat vs. placebo group (*p* = 0.004)	
Tanaka et al., 2015 [25]	-Hyperuricemic (uric acid ≥ 7.0 mg/dL) stage 3 CKD patients -Exclusion criteria: acute/chronic inflammatory disease and/or malignancy, active gout, severe CV/respiratory/digestive disease within 6 months before study entry, pregnancy, medication with Febuxostat and/or Benzbromarone within 3 months before, immunosuppressive therapy	-*N* = 45 -Men (%) = ~87.5 -Age (yr) = ~68 -BMI (kg/m^2^) = ~25 -SBP (mmHg) = ~129 -DBP (mmHg) = ~78 -Diabetic nephropathy (%) = ~8 -Hypertension (%) = ~42 -Uric acid (mg/dL) = ~8.0 -SCr (mg/dL) = ~1.3 -eGFR(mL/min/1.73 m^2^) = ~44.6 -UPCR (g/g) = ~0.67 -UACR (mg/g) = ~78	3 months	Febuxostat, 40 mg/day (*N* = 25)	Standard therapy (*N* = 20)	SCr (mg/dL)	-No difference between groups	-Open label -Febuxostat was administered at an initial dose of 10 mg/d and up-titrated to 40 mg -2 patients in Febuxostat group withdrew due to rash and hypotension. -One patient in the control group and 2 patients in the Febuxostat group were lost to follow-up -21 patients in the Febuxostat and 19 in the control group were analysed after follow-up
						eGFR (mL/min/1.73 m^2^)	-End of treatment, mean change −1.3 ± 4.0 vs. −0.4 ± 5.8 in Febuxostat vs. control group (*p* = 0.59)	
						UPCR (g/g)	End of treatment, mean change −0.36 ± 0.66 vs. 0.07 ± 0.38 in Febuxostat vs. control group (*p* = 0.018)	
						UACR (mg/g)	End of treatment, median change -25.3 (−357.0, 4.8) vs. +5.2 (−71.4, 105.5) in Febuxostat vs. control group (*p* = 0.035)	
Beddhu et al., 2016 [26]	-Overweight or obese adults with hyperuricemia and type 2 diabetic nephropathy -Exclusion criteria: history of gout, concurrent use of Azathioprine, Mercaptopurine, Theophylline, Allopurinol or Warfarin, recent antibiotic therapy, pregnancy, active malignancy, active AIDS, chronic lung disease	-*N* = 80 -Men (%) = 65 -Age (yr) = 68 ± 10 -BMI (kg/m^2^) = 34.6 ± 6.8 -SBP (mmHg) = 127 ± 17 -DBP (mmHg) = 70 ± 12 -Hypertension (%) = 77.5 -Uric acid (μmol/L) = 426 ± 83 -eGFR(mL/min/1.73 m^2^) = 53.5 ± 17.2 -UACR (mg/mmol) = ~2.19 (median)	6 months	Febuxostat, 80 mg/day (*N* = 40)	Placebo (*N* = 40)	eGFR (mL/min/1.73 m^2^)	No difference between groups	-Double blind -One patient in the placebo and 3 in the Febuxostat group withdrew from the study -ITT analysis performed
						UACR (mg/mmol)	-End of treatment, median 1.07 (IQR 0.46, 6.99) vs. 1.15 (IQR 0.42, 7.10) in Febuxostat vs. placebo	
Saag et al., 2016 [27]	-Hyperuricemic, gouty patients with moderate-to-severe CKD -Exclusion criteria: secondary hyperuricemia, xanthinuria, tophaceous gout, use of Aspirin >325 mg/day within 35 days prior to randomization, Allopurinol, Febuxostat or Colchicine hypersensitivity, CV disease, dialysis, liver disease, alcoholism	-*N* = 96 -Men (%) = 80.2 -Age (yr) = 65.7 ± 10.57 -BMI (kg/m^2^) = 33.4 ± 6.67 -Hypertension (%) =95.8 -DM (%) = 44.8 -Uric acid (mg/dL) = 10.5 ± 1.7	12 months	Febuxostat, 30 mg/twice daily (*N* = 32) Febuxostat, 40/80 mg/day (*N* = 32)	Placebo (*N* = 32)	SCr (mg/dL)	No difference between Febuxostat groups and the placebo	-Double blind -At study screening, any urate-lowering therapies were discontinued -SCr levels of ≥1.5 mg/dL occurred in 41% of patients receiving 30 mg Febuxostat, 50% of patients receiving 40/80 mg Febuxostat and 53% of patients receiving placebo -Efficacy and safety analyses performed by the last-observation-carried-forward method
						eGFR (mL/min/1.73 m^2^)	No difference between Febuxostat groups and the placebo	

**Legend:** ACEIs: angiotensin converting enzyme inhibitors, AEs: adverse events, AIDS: Acquired Immune Deficiency Syndrome, ARBs: angiotensin receptor blockers, BMI: body mass index, CKD: chronic kidney disease, CrCl: creatinine clearance, CV: cardiovascular, DBP: diastolic blood pressure, DM: diabetes mellitus, EF: ejection fraction, eGFR: estimated glomerular filtration rate, HbA1c: glycated haemoglobin, HIV: human immunodeficiency virus, IgAN: IgA nephropathy, IQR: interquartile range, ITT: intention-to-treat, LVF: left ventricular failure, LVH: left ventricular hypertrophy, SBP: systolic blood pressure, SCr: serum creatinine, UACR: urine albumin creatinine ratio, UPCR: urine protein creatinine ratio, * main study.

**Table 2:** Risk of bias in randomized controlled trials.

Study, Year (Ref)	Random Sequence Generation	Allocation Concealment	Blinding of Participants and Personnel	Blinding of Outcome Assessors	Incomplete Outcome Data	Selective Reporting	Other Sources of Bias
Siu et al., 2006 [13]	Low risk (randomization performed using a computer-generated list)	Unclear (not stated)	High Risk (open label)	Unclear (not stated)	Low risk (3 drop-outs; per-protocol analysis performed)	Low risk	None known
Momeni et al., 2010 [14]	Unclear (not stated)	Unclear (not stated)	Low Risk (double blind)	Unclear (not stated)	Unclear (not stated)	Low risk	None known
Kao et al., 2011 [15]	Unclear (not stated)	Unclear (not stated)	Low Risk (double blind)	Unclear (not stated)	High risk (overall 14 drop-outs; 15% vs. 25% in intervention vs. control. Per-protocol analysis performed)	Low risk	None known
Shi et al., 2012 [16]	Low risk (“randomization performed using a computer-generated random allocation sequence table”)	Low risk (“allocation was concealed by enclosing assignments in sequentially numbered, opaque-closed envelopes”)	High Risk (open label)	Unclear (not stated)	Low risk (5 drop-outs; ITT analysis performed)	Low risk	None known
Hosoya et al., 2014 * [17] Hara et al., 2015 Jomori et al., 2015	Unclear (not stated)	Unclear (not stated)	Low Risk (double blind)	Unclear (not stated)	Low risk (11 drop-outs; ITT analysis performed)	Low risk	High risk of funding bias (study was funded by Sanwa Kagaku Kenkyusho Co., Ltd. (SKK)
Kim et al., 2014 [18]	Unclear (not stated)	Unclear (not stated)	Low Risk (double blind)	Unclear (not stated)	Low risk (7 drop-outs; last-observation-carried forward analysis performed	Low risk	None known
Sezer et al., 2014 [19]	Unclear (not stated)	Unclear (not stated)	High Risk (open label)	Unclear (not stated)	Unclear (not stated)	Low risk	None known
Goicoechea et al., 2015 * [20] Goicoechea et al., 2010 [21]	Low risk (randomization performed using a computer-generated list)	Unclear (not stated)	High Risk (single blind)	High Risk	Low risk (13 drop-outs; ITT analysis performed)	Low risk	None known
Bayram et al., 2015 [22]	High risk (“patients were randomized in a consecutive manner”)	Unclear (not stated)	High Risk (open label)	Unclear (not stated)	Unclear (not stated)	Low risk	None known
Ivanov and Ivanova, 2015 [23]	Unclear (not stated)	Unclear (not stated)	High Risk (open label)	Unclear (not stated)	Unclear (not stated)	Low risk	None known
Sircar et al., 2015 [24]	Low risk (randomization performed using a computer-generated random-number table)	Low risk (“allocation concealment was done by sealed sequentially numbered opaque envelopes”)	Low Risk (double blind)	Low risk (treatment assigned was not known by the investigator)	Low risk (10 drop-outs; per-protocol analysis performed)	Low risk	Low risk of funding bias (“drugs and placebo were provided by Intas Pharmaceuticals, which had no other role in funding, study design, data collection and analysis, decision to publish or preparation of the manuscript”)
Tanaka et al., 2015 [25]	High risk	High Risk (“simple randomization was used by drawing a sealed envelope containing the intervention allocation from a box”)	High Risk (open label)	High Risk (open label)	Low risk (5 drop-outs; per-protocol analysis performed)	Low risk	None known
Beddhu et al., 2016 [26]	Low risk (“randomization performed by blocks of 4 using a random number generator”)	Unclear (not stated)	Low Risk (double blind)	Low risk (“investigators and study staff were blinded to the treatment assignment”)	Low risk (4 drop-outs; ITT analysis performed)	Low risk	Low risk of funding bias (“the study was funded by a grant from Takeda Pharmaceuticals USA, Inc. The sponsor had no role in the design and conduct of the study or analysis and interpretation of results or preparation of the manuscript”)
Saag et al., 2016 [27]	Unclear (not stated)	Low risk (“Febuxostat and placebo tablets were overencapsulated in a similar manner to ensure blinding of study medication”)	Low Risk (double blind)	Unclear (not stated)	Low risk (efficacy and safety analyses performed by last observation carried forward method)	Low risk	High risk of funding bias (“the study was funded by Takeda Pharmaceuticals, Deerfield, IL. The sponsor authors were involved in the design and conduct of the study, all study analyses, the drafting and editing of the manuscript”)

**Legend:** ITT: intention-to-treat, * main study.

**Table 3:** Summary of findings (GRADE).

Xanthine Oxidase Inhibitors versus Placebo or Standard Therapy			
**Patient or population:** people with chronic kidney disease **Intervention:** Allopurinol, Febuxostat or Topiroxostat **Comparison:** placebo or standard therapy			
**Outcome**	**Effect Estimate** **(95% CI)**	**N. of Participants (Studies)**	**Quality of the Evidence** **(GRADE)**
ESKD	RR 0.42 (0.22,0.80)	204 (3 studies)	⊕⊕⊕⊕ High
Serum Creatinine	MD −0.05 (−0.12,0.02)	270 (3 studies)	⊕⚪⚪⚪^1^ Very Low
eGFR (all studies) (F.U. > 3 mo.) (blind design)	MD 2.33 (−0.27,4.92) MD 6.82 (3.50,10.15) MD 2.61 (0.23,4.99)	641 (7 studies) 357 (4 studies) 400 (3 studies)	⊕⚪⚪⚪^1^ Very Low ⊕⊕⊕⚪^2^ Moderate ⊕⊕⚪⚪^3^ Low
Proteinuria	SMD −0.06 (−0.39,0.26)	191 (4 studies)	⊕⊕⊕⊕ High
Albuminuria *	N/A	303 (4 studies)	N/A

**GRADE Working Group grades of evidence. High quality**: Further research is very unlikely to change our confidence in the estimate of effect. **Moderate quality**: Further research is likely to have an important impact on our confidence in the estimate of effect and may change the estimate. **Low quality**: Further research is very likely to have an important impact on our confidence in the estimate of effect and is likely to change the estimate. **Very low quality**: We are very uncertain about the estimate. ESKD: end-stage kidney disease; GFR: glomerular filtration rate; MD: mean difference; SMD: standardized mean difference; RR: risk ratio; * data from single studies and/or reported in a narrative way (outcome ungradable); 1: Downgraded for high inconsistency and indirectness (applicability in study population/intervention/follow-up/study design); 2: Downgraded for indirectness (applicability in study intervention); 3: Downgraded for inconsistency and indirectness (applicability in study intervention).

## Supplementary Materials

Supplementary materials can be found at www.mdpi.com/1422-0067/18/11/2283/s1.

## References

[1] Bolignano D., Pisano A., Coppolino G. (2016). The Dark Side of Blocking RAS in Diabetic Patients with Incipient or Manifested Nephropathy. Exp. Clin. Endocrinol. Diabetes.

[2] Kumagai T., Ota T., Tamura Y., Chang W.X., Shibata S., Uchida S. (2017). Time to target uric acid to retard CKD progression. Clin. Exp. Nephrol..

[3] Johnson R.J., Nakagawa T., Jalal D., Sanchez-Lozada L.G., Kang D.H., Ritz E. (2013). Uric acid and chronic kidney disease: Which is chasing which?. Nephrol. Dial. Transplant..

[4] Thurston M.M., Phillips B.B., Bourg C.A. (2013). Safety and efficacy of allopurinol in chronic kidney disease. Ann. Pharmacother..

[5] Kabul S., Shepler B. (2012). A review investigating the effect of allopurinol on the progression of kidney disease in hyperuricemic patients with chronic kidney disease. Clin. Ther..

[6] Kang D.H., Nakagawa T., Feng L., Watanabe S., Han L., Mazzali M., Truong L., Harris R., Johnson R.J. (2002). A role for uric acid in the progression of renal disease. J. Am. Soc. Nephrol..

[7] Mazzali M., Hughes J., Kim Y.G., Jefferson J.A., Kang D.H., Gordon K.L., Lan H.Y., Kivlighn S., Johnson R.J. (2001). Elevated uric acid increases blood pressure in the rat by a novel crystal-independent mechanism. Hypertension.

[8] Sanchez-Lozada L.G., Tapia E., Soto V., Avila-Casado C., Franco M., Wessale J.L., Zhao L., Johnson R.J. (2008). Effect of febuxostat on the progression of renal disease in 5/6 nephrectomy rats with and without hyperuricemia. Nephron Physiol..

[9] Bose B., Badve S.V., Hiremath S.S., Boudville N., Brown F.G., Cass A., de Zoysa J.R., Fassett R.G., Faull R., Harris D.C. (2014). Effects of uric acid-lowering therapy on renal outcomes: A systematic review and meta-analysis. Nephrol. Dial. Transplant..

[10] Kanji T., Gandhi M., Clase C.M., Yang R. (2015). Urate lowering therapy to improve renal outcomes in patients with chronic kidney disease: Systematic review and meta-analysis. BMC Nephrol..

[11] Fleeman N., Pilkington G., Dundar Y., Dwan K., Boland A., Dickson R., Anijeet H., Kennedy T., Pyatt J. (2014). Allopurinol for the treatment of chronic kidney disease: A systematic review. Health Technol. Assess..

[12] Filiopoulos V., Hadjiyannakos D., Vlassopoulos D. (2016). Febuxostat Renoprotection in CKD Patients With Asymptomatic Hyperuricemia. Am. J. Kidney Dis. Off. J. Natl. Kidney Found..

[13] Siu Y., Leung K., Tong M., Kwan T. (2006). Use of allopurinol in slowing the progression of renal disease through its ability to lower serum uric acid level. Am. J. Kidney Dis..

[14] Momeni A., Shahidi S., Seirafian S., Taheri S., Kheiri S. (2010). Effect of allopurinol in decreasing proteinuria in type 2 diabetic patients. Iran. J. Kidney Dis..

[15] Kao M., Ang D., Gandy S., Nadir M., Houston J., Lang C., Struthers A. (2011). Allopurinol benefits left ventricular mass and endothelial dysfunction in chronic kidney disease. J. Am. Soc. Nephrol..

[16] Shi Y., Chen W., Jalal D., Li Z., Chen W., Mao H., Yang Q., Johnson R.J., Yu X. (2012). Clinical outcome of hyperuricemia in IgA nephropathy: A retrospective cohort study and randomized controlled trial. Kidney Blood Press. Res..

[17] Hosoya T., Ohno I., Nomura S., Hisatome I., Uchida S., Fujimori S., Yamamoto T., Hara S. (2014). Effects of topiroxostat on the serum urate levels and urinary albumin excretion in hyperuricemic stage 3 chronic kidney disease patients with or without gout. Clin. Exp. Nephrol..

[18] Kim H.A., Seo Y.I., Song Y.W. (2014). Four-week effects of allopurinol and febuxostat treatments on blood pressure and serum creatinine level in gouty men. J. Korean Med. Sci..

[19] Sezer S., Karakan S., Atesagaoglu B., Acar F.N. (2014). Allopurinol reduces cardiovascular risks and improves renal function in pre-dialysis chronic kidney disease patients with hyperuricemia. Saudi J. Kidney Dis. Transplant..

[20] Goicoechea M., Garcia de Vinuesa S., Verdalles U., Verde E., Macias N., Santos A., Perez de Jose A., Cedeno S., Linares T., Luno J. (2015). Allopurinol and progression of CKD and cardiovascular events: Long-Term follow-up of a randomized clinical trial. Am. J. Kidney Dis..

[21] Goicoechea M., de Vinuesa S.G., Verdalles U., Ruiz-Caro C., Ampuero J., Rincon A., Arroyo D., Luno J. (2010). Effect of allopurinol in chronic kidney disease progression and cardiovascular risk. Clin. J. Am. Soc. Nephrol..

[22] Bayram D., Tuqrul S.M., Inal S., Altunta A., Kidir V., Orhan H. (2015). The effects of allopurinol on metabolic acidosis and endothelial functions in chronic kidney disease patients. Clin. Exp. Nephrol..

[23] Ivanov D., Ivanova M. (2015). Febuxostat improves GFR and BP in non-diabetic adults with CKD 2–3: 4 years follow-up. Nephrol. Dial. Transplant..

[24] Sircar D., Chatterjee S., Waikhom R., Golay V., Raychaudhury A., Chatterjee S., Pandey R. (2015). Efficacy of Febuxostat for Slowing the GFR Decline in Patients With CKD and Asymptomatic Hyperuricemia: A 6-Month, Double-Blind, Randomized, Placebo-Controlled Trial. Am. J. Kidney Dis..

[25] Tanaka K., Nakayama M., Kanno M., Kimura H., Watanabe K., Tani Y., Hayashi Y., Asahi K., Terawaki H., Watanabe T. (2015). Renoprotective effects of febuxostat in hyperuricemic patients with chronic kidney disease: A parallel-group, randomized, controlled trial. Clin. Exp. Nephrol..

[26] Beddhu S., Filipowicz R., Wang B., Wei G., Chen X., Roy A.C., DuVall S.L., Farrukh H., Habib A.N., Bjordahl T. (2016). A Randomized Controlled Trial of the Effects of Febuxostat Therapy on Adipokines and Markers of Kidney Fibrosis in Asymptomatic Hyperuricemic Patients With Diabetic Nephropathy. Can. J. Kidney Health Dis..

[27] Saag K., Whelton A., Becker M.A., MacDonald P., Hunt B., Gunawardhana L. (2016). Impact of Febuxostat on Renal Function in Gout Patients With Moderate-to-Severe Renal Impairment. Arthritis Rheumatol..

[28] Jalal D.I., Chonchol M., Chen W., Targher G. (2013). Uric acid as a target of therapy in CKD. Am. J. Kidney Dis..

[29] Yelken B., Caliskan Y., Gorgulu N., Altun I., Yilmaz A., Yazici H., Oflaz H., Yildiz A. (2012). Reduction of uric acid levels with allopurinol treatment improves endothelial function in patients with chronic kidney disease. Clin. Nephrol..

[30] Sanchez-Lozada L.G., Tapia E., Santamaria J., Avila-Casado C., Soto V., Nepomuceno T., Rodriguez-Iturbe B., Johnson R.J., Herrera-Acosta J. (2005). Mild hyperuricemia induces vasoconstriction and maintains glomerular hypertension in normal and remnant kidney rats. Kidney Int..

[31] Vargas-Santos A.B., Neogi T. (2017). Management of Gout and Hyperuricemia in CKD. Am. J. Kidney Dis..

[32] GRADE Working Group (2004). Grading quality of evidence and strength of recommendations. BMJ.

[33] Moher D., Liberati A., Tetzlaff J., Altman D.G. (2009). Preferred reporting items for systematic reviews and meta-analyses: The PRISMA statement. PLoS Med..

[34] Bolignano D., Pisano A., D’Arrigo G. Xanthine oxidase inhibitors for improving renal damage in CKD patients. http://www.crd.york.ac.uk/PROSPERO/display_record.asp?ID=CRD42017067881.

[35] National Kidney Foundation (2002). K/DOQI clinical practice guidelines for chronic kidney disease: Evaluation, classification, and stratification. Am. J. Kidney Dis..

[36] Higgins J.P., Thompson S.G., Deeks J.J., Altman D.G. (2003). Measuring inconsistency in meta-analyses. BMJ.

