# Supplementary material for: Xanthine Oxidase Inhibitors for Improving Renal Function in Chronic Kidney Disease Patients: An Updated Systematic Review and Meta-Analysis
Source: Int J Mol Sci. 2017 Oct 31;18(11):2283. doi: 10.3390/ijms18112283 (PMC5713253; doi:10.3390/ijms18112283)
Supplement: Supplementary file 1 [file ijms-18-02283-s001.pdf]

**Table S1.** Search strategy in CENTRAL, Ovid-Medline and PubMed databases.

| **CENTRAL** |
|---|
| #1 xanthine oxidase inhibitors<br>#2 MeSH descriptor: [Gout Suppressants] explode all trees<br>#3 Febuxostat<br>#4 MeSH descriptor: [Allopurinol] explode all trees<br>#5 Allopurinol<br>#6 MeSH descriptor: [Febuxostat] explode all trees<br>#7 Topiroxostat<br>#8 MeSH descriptor: [Kidney Failure, Chronic] explode all trees<br>#9 MeSH descriptor: [Renal Insufficiency, Chronic] explode all trees<br>#10 CKD or CRF or CRI<br>#11 renal insufficiency or renal failure<br>#12 (#1 or #2 or #3 or #4 or #5 or #6 or #7)<br>#13 (#8 or #9 or #10 or #11)<br>#14 (#12 and #13) |
| **OVID-Medline** |
| 1. Kidney diseases [Included related terms]<br>2. Kidney diseases.mp. or exp Kidney Diseases/<br>3. Renal insufficiency.mp. or exp Renal Insufficiency/<br>4. Chronic renal insufficiency.mp. or exp Renal Insufficiency, Chronic/<br>5. chronic kidney.tw.<br>6. chronic renal.tw.<br>7. CKF.tw.<br>8. ckd.tw.<br>9. crf.tw.<br>10. crd.tw.<br>11. 1 or 2 or 3 or 4 or 5 or 6 or 7 or 8 or 9 or 10<br>12. exp Febuxostat/ or exp Allopurinol/ or exp Gout Suppressants/<br>13. febuxostat.tw.<br>14. allopurinol.tw.<br>15. topiroxostat.tw.<br>16. 12 or 13 or 14 or 15<br>17. 11 and 16<br>18. limit 17 to human |
| **PubMed** |
| ((xanthine oxidase inhibitor OR topiroxostat OR allopurinol OR febuxostat) AND (chronic renal insufficiency OR chronic kidney disease OR chronic renal failure OR CKD or CRF)) |

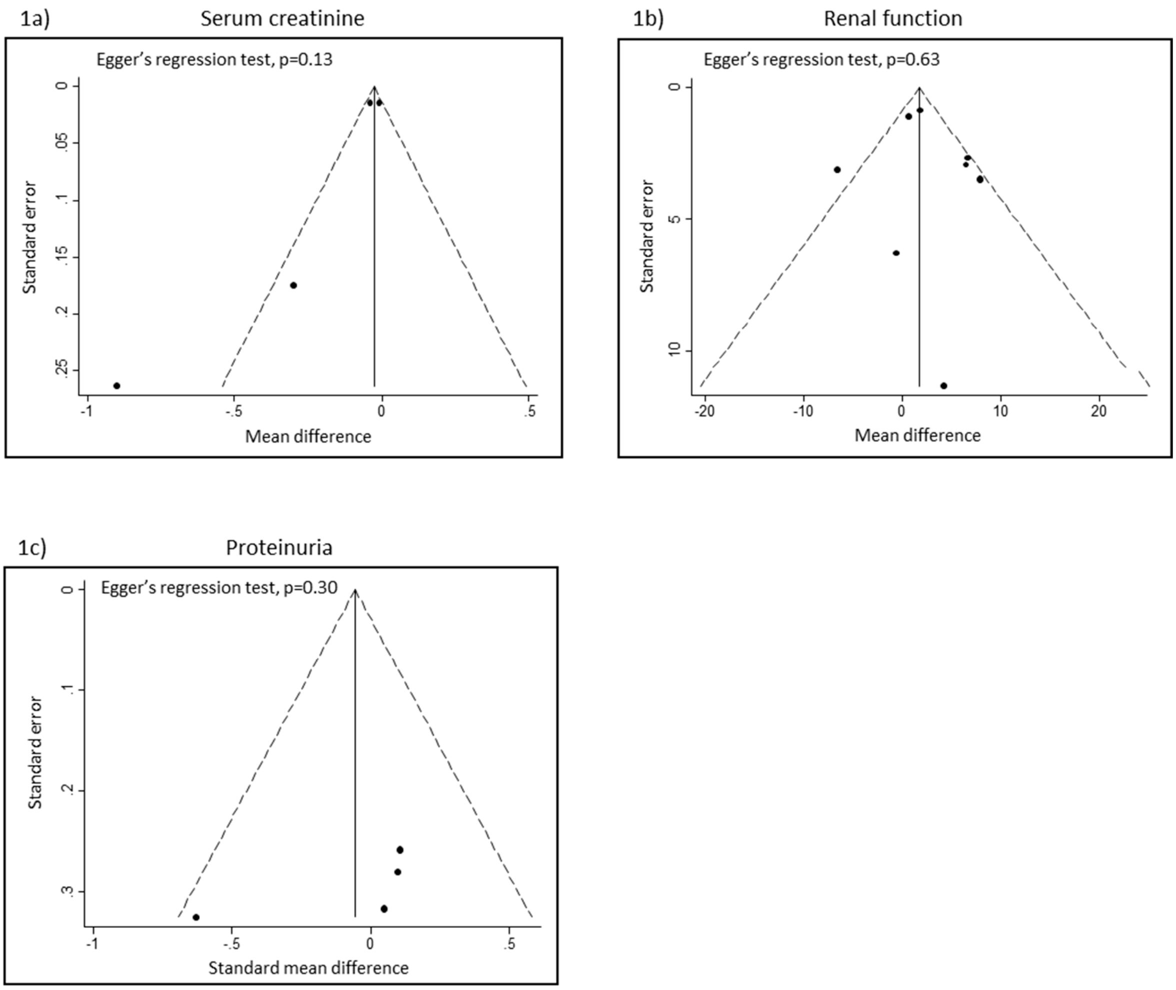


**Figure S1.** Funnel plots of standard error by difference/standard difference in means and Egger's regression test for assessing publication bias
